# Recent Advances in Liver Resection for Hepatocellular Carcinoma

**DOI:** 10.3389/fsurg.2014.00021

**Published:** 2014-06-16

**Authors:** Zenichi Morise, Norihiko Kawabe, Hirokazu Tomishige, Hidetoshi Nagata, Jin Kawase, Satoshi Arakawa, Rie Yoshida, Masashi Isetani

**Affiliations:** ^1^Department of Surgery, Banbuntane Houtokukai Hospital, Fujita Health University School of Medicine, Nagoya, Japan

**Keywords:** hepatocellular carcinoma, liver resection, laparoscopic hepatectomy, chemotherapy, 3-dimensional computed tomography

## Abstract

Hepatocellular carcinoma (HCC) is the most common primary liver malignancy. The association of HCC with chronic liver disease (CLD) is well known and making treatment complex and challenging. The treatment of HCC must take into consideration, the severity of CLD, the stage of HCC, and the clinical condition of the patient. Liver resection (LR) is one of the most efficient treatments for patients with HCC. Better liver function assessment, increased understanding of segmental liver anatomy using more accurate imaging studies, and surgical technical progress are the important factors that have led to reduced mortality, with an expected 5 year survival of 38–61% depending on the stage of the disease. However, the procedure is applicable to <30% of all HCC patients, and 80% of the patients after LR recurred within 5 years. There are recent advances and prospects in LR for HCC in several aspects. Three-dimensional computed tomography imaging assisted preoperative surgical planning facilitates unconventional types of LR. Emerging evidences of laparoscopic hepatectomy and prospects for the use of newly developing chemotherapies as a combined therapy may lead to expanding indication of LR. LR and liver transplantation could be associated rather than considered separately with the current concepts of “bridging LR” and “salvage transplantation.”

## Introduction

Hepatocellular carcinoma (HCC) is the sixth most common cancer and the most common primary liver malignancy (1). The association of HCC with chronic liver disease (CLD), due to viral infection, alcohol consumption, metabolic syndrome, etc., is well known and making treatment complex and challenging. The underlying liver parenchyma displays various histological changes, including steatosis, inflammation, and fibrosis to cirrhosis. These histological changes of the underlying parenchyma and the risk of multicentric carcinogenesis from chronically injured liver tissue limit the possibility of curative treatments, which include local ablation of small size and number tumors, liver resection (LR), and liver transplantation (LT) (2).

Liver resection is one of the most efficient treatments for HCC patients (3, 4). Considerable progress observed during the last 10 years in screening, treatment of the underlying liver disease, early radiological diagnosis, and surgical techniques has updated the indications for LR (2). Better liver function assessment, understanding of segmental liver anatomy using more accurate imaging studies, and surgical technical progress are the most important factors that have led to reduced mortality, with an expected 5-year survival of 38–61% depending on the stage of the disease (5). However, only <30% of patients with HCC are eligible for surgery (3, 4). Emerging evidences of laparoscopic hepatectomy and prospects for the use of newly developing chemotherapies as a combined therapy may lead to expanding indication of LR (6, 7).

In this review, we present recent advances in LR of HCC.

## Current Concept of Liver Resection for Hepatocellular Carcinoma

The largest report of LR for HCC is from the Liver Cancer Study Group in Japan, which has reported 1-, 3-, 5-, and 10-year survival rates of 85, 64, 45, and 21%, respectively, in 6,785 cirrhotic patients treated by LR between 1988 and 1999 (8). Comparable results have been reported by other groups worldwide without differences between Western and Eastern countries. Survival rates as high as 60% at 5 years could have been achieved in Child-Pugh A patients with well-encapsulated tumors of ≤2 cm in diameter. Results from patients with good liver function and anatomical LR according to the architecture of the portal vein (although <10% of all patients) could be favorably compared with those from patients with LT.

There are several reports describing that anatomical LR for small solitary HCC achieve significantly better overall and disease-free survival than limited resections, without increasing the post-operative risk (9, 10). Intrahepatic metastasis of HCC along the portal vein and the presence of satellite nodules within 2 cm from the main nodule (11) are the basis of the concept for anatomical LR, complete removal of the tumor-bearing portal territory. Anatomical LR has the potential to remove undetected cancerous foci (portal vein metastases and satellite nodules) disseminated from the main tumor and is recommended in many reports if possible.

In patients with HCC, both tumor extension and severity of liver dysfunction influence the indication and extent of LR. When considering the treatment of HCC in patients with CLD, the degree of invasive surgical stress, especially to the impaired liver, should be considered in addition to the oncological therapeutic effects. Patients with severe CLD have various (overt and preliminary) symptoms, such as (1) deteriorations of protein synthesis and metabolism; (2) GI tract congestion, ascites, and pancytopenia due to portal hypertension and hypersplenism; and (3) susceptibility to infectious diseases and hepatopulmonary syndrome (hypoxemia) due to increased shunt vessels (12). Cirrhotic patients have high morbidity and mortality following anesthesia and surgery (13), and the risk of abdominal operations increases according to the preoperative Child-Pugh classification (14) of the patients (15). Major histological changes observed in patients with HCC include fibrosis ranging from mild (F1) to cirrhosis (F4). Latter stages of cirrhosis also have a lower rate of regeneration after LR, more frequent association with portal hypertension, and higher risk of tumor multiplicity/recurrence (16, 17). Steatosis and the inflammatory process also have a significant influence on the course after LR, even absence of extensive fibrosis. LR in diseased parenchyma presents operative risk due to altered texture of the liver parenchyma, impaired liver regeneration, and deteriorated liver function leading to coagulation defects, an increased risk of infection, etc. (18). There is a close relationship between the extent of resected liver volume and post-operative morbidity/mortality of LR in patients with CLD. The fact limits the indication of LR for large tumors or small but centrally located tumors (19). The most difficult point of LR in patients with HCC and CLD is that it should be curative with the resection of the tumor vascular territories and also preserve as much liver volume as possible to prevent post-operative liver failure.

## Assessment and Modulation of Remnant Liver Function

Small remnant liver volume is associated with poor post-operative liver function and a high mortality/morbidity after LR (20). Although the safety limit for the remnant liver volume in patients with normal liver is approximately 30% of the total liver volume (TLV), it is generally thought that a remnant liver volume of 40–50% should be preserved after major LR in patients with CLD. The liver is characterized by its capacity to ensure apparent normal liver function with a reduced functional volume and its ability to regenerate. However, the ability to regeneration varies depending on factors such as fibrosis of remnant liver, portal flow, etc. Thus, adequate volume of future liver remnant (FLR) should be different in each individual patient. Although the aim of preoperative assessment of liver function is to prevent post-operative liver failure in each individual patient, the assessments for the post-operative functionality of a reduced-volume FLR and its capacity to regenerate is difficult. Because there are no valuable stress tests to assess the potential of liver function, preoperative assessment in patients with CLD involves joint interpretation of several biological, morphological, histological, and hemodynamic factors.

One widely used method of biological assessment is the Child-Pugh classification (11), which was originally designed for predicting the prognosis of patients with portal hypertension undergoing shunting operations. Resection is contraindicated in grade C cirrhotic patients and restricted to very limited resection in grade B cirrhotic patients (21). However, even in grade A cirrhotic patients with apparently normal liver function, the risk of liver surgery is increased, necessitating the development of more sophisticated quantitative liver function tests. Among the various methods available, the indocyanine green (ICG) clearance rate represents the most common test for predicting mortality after hepatectomy (22, 23). The normal ICG value in healthy patients is approximately 10%, and cutoff values predictive of safe major hepatectomies range from 14 to 17% (24, 25). Minor resections can be performed for values up to 22% (26), limited hepatectomies for values up to 40% (19), and limited wedge laparoscopic resections, in the opinion of some researchers, can be tolerated for even higher values (27, 28). The model for end-stage liver disease (MELD) score was validated as an accurate predictor of survival among different populations of patients with advanced liver disease (29, 30). In the case of LR, the impact of the MELD score was studied only retrospectively in some series of cirrhotic patients, who had undergone LR for HCC. In two series of cirrhotic patients who underwent LR for HCC, the authors showed that a MELD score >8 was associated with a higher risk of mortality, morbidity, and impaired long-term survival (29, 30).

Preoperative portal vein embolization (PVE) was first introduced by Makuuchi et al. (28) and widely recognized as an effective method for preoperative volume modulation for small FLR. However, the degree of hypertrophy of the FLR after PVE is variable in patients with CLD (20, 31). It is generally accepted that the absence of early hypertrophy of a non-embolized liver following technically successful PVE is an indicator of low regenerative capacity that would contraindicate LR, and thus, the response represents a real dynamic stress test before major LR (32). It has been shown that sequential selective transarterial chemoembolization (TACE) before PVE increases the rate of hypertrophy (32, 33). In the event of inadequate FLR hypertrophy that precludes LR, this combined vascular obstruction of the tumor territory represents an efficient treatment of HCC. As other means of anticipating post-operative liver failure, there are several reports using the volumetry data from computed tomography (CT) for the evaluations of the FLR volume in the proportion of body weight, body surface area, and TLV (34, 35), the hypertrophy rate of the FLR/TLV ratio (36), etc.

## Anatomical Resection and Imaging

The anatomical territory of HCC ranges from subsegment to lobe according to the size and location of the tumor. Anatomical resections of small solitary HCCs achieve significantly better overall and disease-free survival than do limited resections, without increasing the post-operative risk (9, 10). However, the benefit of segmental resection may only become apparent in tumors between 2 and 5 cm. For the tumors <2 cm in size, the risk of dissemination is considered to be negligible with equivalent efficacy of local ablative therapy; for the tumors more than 5 cm, majority of patients will already have macroscopic vascular invasion or satellite nodules that lead to a high incidence of recurrence (37). In the case of central tumors with undefined vascular territory, some authors have found lower recurrence rates and greater survival with 2-cm surgical margins compared with 1-cm margins (38), and other authors have found no difference between margins <1 or >1 cm (39, 40). The adequate margin of LR should also depend on the tumor type (with/without capsules, with/without invasion outside the capsule, etc.) and still under discussion. Three-dimensional CT-assisted preoperative surgical planning allows determination of resectability and changes in operative strategy (resection modifications/extensions/intrahepatic vascular reconstructions studying portal distribution, hepatic vein anatomy for adequate venous drainage, biliary distribution for avoiding biliary fistula) (41). This preoperative assessment seems particularly helpful in patients with unconventional resection planes and in those with central tumors. It allows complicated anatomical LR being adapted to more number of patients, such as subsegment anatomical LR for small tumor in highly injured liver and anatomical LR of combined territories for deep centrally located tumor on the border of territories.

## Laparoscopic LR

Since the first successful report in 1992 (42), laparoscopic LR is thought to be a less invasive procedure than conventional open LR for the treatment of hepatic lesions (43). In a comprehensive meta-analysis study, laparoscopic LR was compared to open in 1,678 patients across 26 studies (44). While it is associated with longer operating times and no differences in oncological outcomes, it is advantageous in several aspects. Laparoscopic LR is associated with reduced blood loss, decreased portal clamp time, decreases in overall and liver-specific complications, and shorter post-operative hospital stays. Recent technological development of devices and accumulation of experiences have facilitated the expansion of the indication of the procedure (6, 45). Although common advantages of laparoscopic surgery, such as early recovery and discharge with smaller post-operative pain and earlier intake, have also been reported for laparoscopic hepatectomy (46), specific advantages and the indication of laparoscopic LR have yet to be well settled.

Even limited LR under open surgery for severe CLD patients often develops refractory ascites, which leads to fatal complications (47, 48). Laparoscopic LR may be particularly advantageous for those patients, given the potential for lower levels of parietal and hepatic injury and the preservation of venous and lymphatic collateral circulation, especially for the patients with multicentric/metachronous lesions occurring in chronically injured liver. The safety and feasibility of the laparoscopic approach and its short-term benefits for HCC patients with CLD have been demonstrated by several series. To date, several studies have investigated the major differences between laparoscopic LR and open LR (49–59). Favorable short-term results, including fewer incidences of ascites and liver failure, and shorter post-operative hospital stay correlates with the laparoscopic procedure. Tranchart et al. reported laparoscopic LR of HCC for selected patients resulted in better post-operative outcomes without long- and short-term oncologic consequences (60). Also in our experience (61), we reported the favorable perioperative course of the patients with severe CLD (Child B/C and ICG R15 of 40% or above) who underwent pure laparoscopic LR, which is comparable to that of the patients with mild/moderate CLD (Child A and ICG R15 of 10.1–27.4%). This study showed that laparoscopic LR has the advantage of minimal ascites (61) in addition to usual advantages of laparoscopic surgery, due to the preservation of venous and lymphatic collateral circulation, which leads to lower risk of disturbance in water and/or electrolyte balance and hypoproteinemia that could trigger fatal liver failure. This feature could be the most remarkable specific advantage for laparoscopic LR. Patients who undergo LR are exposed to three different types of stresses: (1) general, whole-body surgical stress, (2) reduced liver function due to resected liver volume, and (3) surgery-induced injury for liver parenchyma and environment around the liver caused by destruction of the collateral blood/lymphatic flow by laparotomy plus mobilization of the liver and parenchymal injury by compression of the liver. Reduction of the third mentioned stress by laparoscopic LR should lower the risk for HCC patients with severe CLD. Among these patients with severe CLD in our series, one underwent living-related LT 20 months after hepatectomy (28). The procedure could also be an advantageous option in bridging therapy to LT for certain HCC patients with severe CLD. Furthermore, our experience showed laparoscopic LR also results in improved vision and manipulation in a small operative field under several conditions, such as repeat hepatectomy with adhesions (28) and, also, some novel approach of LR [for example, caudal approach, not anterior approach, for oncologically appropriate posterior sectorectomy and right hepatectomy (62)] is also possible laparoscopically. These characteristics of laparoscopic LR may indicate that it is a superior method when compared to open LR under certain conditions and its application may lead to expanding indication of LR.

## Adjuvant and/or Combined Therapy for LR

Recurrence after LR occurs in up to 80% of patients at 5 years (63). Two-year cutoff has been raised to distinguish between early and late recurrence. Two-thirds of the recurrence occurs within 2 years, which is considered as dissemination from the original tumor (64). The factors related to this recurrence are tumor size, microvascular invasion, satellite nodules, α-fetoprotein levels, and non-anatomical resection. Large portion of delayed recurrence may correspond to “*de novo*” tumors in the oncogenic CLD liver (65). Presence of cirrhosis (F4), hepatitis activity, and multinodularity are in the risk factors associated with delayed recurrence, besides vascular invasion, and moderate or poorly differentiated HCC (64).

Several strategies have been tested to prevent recurrence, such as preoperative chemoembolization (66), chemotherapy, internal radiation (67), adoptive immunotherapy (68), retinoids (69), or interferon. Three meta-analyses that favored the use of interferon have been published (70, 71), although there are few good-quality studies. Whether there is definite efficacy of the agent and, if there is, also whether the effect of interferon works on early recurrence as an anti-cancerous agent or on delayed recurrence through the control of CLD activity are still under discussion. The efficacy of sorafenib in advanced stages has encouraged evaluation of this agent at earlier phases of the disease, and the trials are ongoing. There is no proven neoadjuvant therapy that can decrease or delay the incidence of intrahepatic recurrence after LR (72). Despite the facts that TACE can downstage HCC, prospective trials have failed to show any significant benefit of this treatment before LR (73, 74). Although recurrence following LR is associated with a poor outcome in most cases, there is growing evidence that some patients with only intrahepatic recurrence will benefit from more aggressive approaches (75, 76). Multimodality therapy of recurrence, including TACE, percutaneous ablative therapy, and re-resection could result in prolonged survival, with an overall 5-year survival rate of 20% (75).

Vascular invasion of HCC, particularly portal and hepatic venous tumor thrombus (VTT), is one of the indicators of patient prognosis and development of tumor thrombi in a major branch of the veins is a frequent terminal feature of HCC. The prognosis of such patients is extremely poor, and survival is limited to a few months after diagnosis (77–79). For these advanced HCCs, conventional therapies like TACE and percutaneous ablative therapy are not indicated due to lack of efficacy and associated complications (80, 81). LT is a contra-indication for such cases (80). Several reports have mentioned the feasibility of LR for patients with VTT. However, the outcome is unsatisfactory with median survival of 6–12 months (77, 78, 81, 82), except for the cases with VTT located in the segmental or sectoral branches (83). Several approaches have been attempted to improve the surgical results, including combined radiotherapy and TACE to date with unsatisfactory results (84–87). There are recently emerging reports that the clinical efficiency of IFN-α plus transarterial 5-fluorouracil (5-FU) combination therapy for advanced HCC with VTT and intrahepatic metastasis (88–90) and also for resectable HCC as a post-operative adjuvant (91) and a multimodal treatment (7). The results show that this combination therapy with LR is a promising candidate for the treatment of such condition and other advanced cases. Now, several clinical trials for this combination therapy are ongoing. We also previously reported the efficacy of S-1 plus cisplatin combination therapy for the patients with advanced and/or recurrent primary liver carcinomas including HCC (92) and of TACE using degradable starch microspheres (DSM) in patients with liver tumors (93). This chemoembolization using DSM induces only short-term temporary vascular occlusion and chemotherapeutic agent retention on the site, which is applicable repeatedly in short interval with mild damage on the vessels and non-cancerous liver tissue (93). The potential candidates of adjuvant and/or combined therapy for LR should be tested for the future improvement of prognosis in recurrent and/or advanced patients.

**Table 1:** **Summary of recent advances in liver resection for hepatocellular carcinoma**.

**1. Current concept of liver resection for hepatocellular carcinoma**
Most available and efficient treatment for HCC Applicable <30% of all HCC patients 5 year survival rate after resection is 38–61% depending on the stages 80% Of the patients recurred within 5 years after resection
**2. Recent advances in liver resection for hepatocellular carcinoma**
3D CT-assisted preoperative surgical planning
Facilitation for unconventional types of liver resection
Laparoscopic liver resection
Benefits for the patients with severe CLD with lower morbidity
Benefits for the repeat resection
(Benefits as a bridging therapy for liver transplantation)
Adjuvant and/or combined therapy with newly developing chemotherapy (sorafenib, intra-arterial 5-FU plus IFN therapy for HCC with VTT, etc.)
Prospects of expanding indication for advanced tumor
Current concepts of “bridging LR” and “salvage transplantation”
LR and LT could be associated rather than considered separately

## Liver Resection and Liver Transplantation

Impairment of liver function and the risk of multicentric carcinogenesis from chronically injured liver tissue lead to consideration of LT as the ideal treatment for removal of existing tumors and injured/preneoplastic underlying liver. Furthermore, it also prevents the development of post-operative complications associated with portal hypertension and liver failure. LT is not limited by liver function, and in selected patients with limited tumors, survival is similar to LT for other indications (94, 95). However, it became evident that patients with extensive HCC had very poor outcomes, whereas most patients with small tumor load could be cured. Thus, there are discussions for the LT in patients with HCC about the matters, such as the selection of patients in the background of the organ shortage, control of tumors for patients on the waiting list (96).

On the waiting list for LT, HCC patients can experience tumor growth beyond the LT criteria and a high cumulative probability of drop-out from the waiting list. This probability has been reported to range between 7 and 11% at 6 months and to be approximately 38% at 12 months after enrollment by two reports at the late 1990s (97, 98).

Belghiti et al. have demonstrated that surgical resection before LT (“bridging LR”) does not increase the surgical risk nor impair survival (99) and stated that resection and transplantation could be associated rather than considered separately. They mentioned that resection could be used as a bridge to transplantation, especially for tumors located in the upper part of the right liver, which can be easily and completely removed through a transthoracic incision. Similarly, some superficial tumors that are not easily accessible by a percutaneous approach could be resected through a laparoscopic approach.

Several studies have confirmed that LT for recurrence after LR did not increase the operative risk and offered a chance of long-term survival when HCC recurrence was limited (99–101). Initial LR of HCC as primary therapy in patients, who otherwise could have been transplanted offers good quality of life and is less demanding than LT. Patients do not need long-term immunosuppression; in addition, grafts are saved for the community and can be transplanted to other patients with no alternative to LT (99, 100, 102). “Salvage transplantation” was first proposed by Majno et al. (103) for tumor recurrence or deterioration in liver function of the patients after LR as primary therapy. This concept seems to be applicable in a significant proportion of patients with long-term survival similar to that of patients who undergo primary LT (99–101). Also, histological analysis of specimens from LR, either in “bridging” or “salvage” setting, allows to show pejorative factors of the tumor, which lead to a valid selection of the subgroup of patients who could benefit from following LT.

## Conclusion (Table 1)

The association of HCC with CLD is making treatment complex and challenging. The treatment of HCC must take into consideration, the severity of CLD, the stage of HCC, and the clinical condition of the patient.

There are recent advances and prospects in LR for HCC in several aspects. Three-dimensional CT imaging assisted preoperative surgical planning facilitates unconventional types of LR. Emerging evidences of laparoscopic hepatectomy and prospects for the use of newly developing chemotherapies as a combined therapy may lead to expanding indication of LR.

Liver resection and LT could be associated rather than considered separately with the current concepts of “bridging LR” and “salvage transplantation.”

## Author Contributions

Zenichi Morise wrote the manuscript; Norihiko Kawabe, Hirokazu Tomishige, Hidetoshi Nagata, Jin Kawase, Satoshi Arakawa, Rie Yoshida, and Masashi Isetani collected the data and assisted in writing of the manuscript.

## Conflict of Interest Statement

We or our institution at any time did not receive any payment or services from a third party for any aspect of the submitted work. There is no financial relationship with entities that could be perceived to influence, or that give the appearance of potentially influencing, what we wrote in the submitted work. There are no patent and copyright, whether pending, issued, licensed, and/or receiving royalties relevant to the work. There is no other relationship or activity that readers could perceive to have influenced, or that give the appearance of potentially influencing, what we wrote in the submitted work.
